# Influence of Restorative Materials on Color of Implant-Supported Single Crowns in Esthetic Zone: A Spectrophotometric Evaluation

**DOI:** 10.1155/2017/5034358

**Published:** 2017-11-19

**Authors:** Min Peng, Wei-Jie Zhao, Mandana Hosseini, Wen-Juan Zhou, Ting Xiao, Jun-Lan Chuan

**Affiliations:** ^1^Department of Stomatology, Sichuan Academy of Medical Sciences and Sichuan Provincial People's Hospital, School of Medicine, University of Electronic Science and Technology of China, Chengdu, Sichuan, China; ^2^Department of Stomatology, The Second People's Hospital of Deyang City, Sichuan, China; ^3^Department of Rehabilitation, School of Dentistry, Faculty of Health Sciences, University of Copenhagen, Copenhagen, Denmark; ^4^Department of Pharmacy, Sichuan Academy of Medical Sciences and Sichuan Provincial People's Hospital, School of Medicine, University of Electronic Science and Technology of China, Chengdu, Sichuan, China

## Abstract

Restorations of 98 implant-supported single crowns in anterior maxillary area were divided into 5 groups: zirconia abutment, titanium abutment, and gold/gold hue abutment with zirconia coping, respectively, and titanium abutment with metal coping as well as gold/gold hue abutment with metal coping. A reflectance spectrophotometer was used to evaluate the color difference between the implant crowns and contralateral/neighboring teeth, as well as the color difference between the peri-implant soft tissue and the natural marginal mucosa. The mucosal discoloration score was used for subjective evaluation of the esthetic outcome of soft tissue around implant-supported single crowns in the anterior zone, and the crown color match score was used for subjective evaluation of the esthetic outcome of implant-supported restoration. ANOVA analysis was used to compare the differences among groups and Spearman correlation was used to test the relationships. A gold/gold hue abutment with zirconia coping was the best choice for an esthetic crown and the all-ceramic combination was the best for peri-implant soft tissue. Significant correlation was found between the spectrophotometric color difference of peri-implant soft tissue and mucosal discoloration score, while no significant correlation was found between the total spectrophotometric color difference of implant crown and crown color match score.

## 1. Introduction 

In the maxillary anterior region, the aesthetic outcome is a critical determinant in the overall success of implant therapy [1–5]. Satisfied esthetic rehabilitation consists of not only natural-looking prosthodontic components but also peri-implant soft tissue characterized by appropriate contour, volume, and color in harmony with the healthy surrounding structures. Thus, the color of both restorative components and peri-implant soft tissue/gingival should be taken into account as crucial esthetical factors [2, 6, 7].

Crown color matching included a series of steps such as color determination, transferring, fabrication, and evaluation, of which the choice of restorative material in fabrication contributes greatly [8]. Translucency is another primary factor in controlling esthetics and it is critical in the selection of materials. Among the large variety of available material gold, titanium, alumina (Al_2_O_3_), and zirconium (ZrO_2_) are the most frequently used material in clinic. The all-ceramic preference is based on an inherent translucency associated with these materials, which allows dentists and lab technicians to fabricate restorations that are like natural teeth. Thus the all-ceramic abutments made of aluminum oxide or yttrium-stabilized zirconium oxide have been produced to meet the need [9]. On the other hand, however, increased thicknesses of alumina and zirconium oxide would compromise the esthetic result due to an increased opacity and reduced translucency [10, 11]. Carossa et al. [12] reported there was no esthetic contraindication using the polished gold alloy posts and cores with all-ceramic crowns. Besides, previous researches focused on the position, inclination, and shape of the restoration regarding the esthetic outcome in implant dentistry, but researches concerned about the influence of restorative material on crown color and translucency were rare, especially by spectrophotometric evaluation.

Additionally, it was reported that the restorations can cause discoloration of the peri-implant soft tissue [11, 13]. The esthetic benefit of ceramic abutments over metal abutments on gingival discoloration has been well documented in clinical studies by Bressan et al. [14, 15]. However, only limited evidence is available concerning the influence of crown material/types on gingival discoloration.

Therefore, the purpose of this clinical trial on implant-supported restorations was to analyze, through spectrophotometric digital technology, the influence of the abutment and crown coping material on color of both implant crown and peri-implant soft tissue. Moreover, the correlation between the spectrophotometric color measurements and the subjective clinical evaluations of color match between the implant crowns and natural teeth and between the marginal peri-implant mucosa and natural gingiva were analyzed.

The null hypothesis of this study was that restoration materials have no effect on the color difference between the implant crowns and natural teeth and between the marginal peri-implant mucosa and natural gingiva.

## 2. Materials and Methods

### 2.1. Spectrophotometric Setup

In the present study, a reflectance spectrophotometer (SpectroShade™, Micro, serial number HDL3214, MHT S.p.A, Verona, Italy) was used, disposing of a D65 light source (6,500 K) that was transformed into monochromatic light (*λ* = 400~720 nm) using a grating. This light was split so that teeth could be illuminated simultaneously from two sides at a 45-degree angle using an intraoral camera. The reflected light was directed at 0 degrees on the system's detector area (18 mm *∗* 14 mm^2^) for a measuring process described previously [6, 16]. One detector is a color CCD chip that generates the color video image. Another black and white CCD detector records the spectrophotometric data. Polarization filters are used to eliminate surface gloss. The data is stored in a proprietary image file format which is used to create detailed CIE *L*^*∗*^*a*^*∗*^*b*^*∗*^ data.

### 2.2. Patients

Fifty-two patients, 39 females and 13 males with a mean age of 25 years (range from 20 to 39 years), were recruited. Patients had either one or two implant-supported single crowns (ISSCs) in the anterior maxillary area because of agenesis. Altogether 98 ISSCs were pooled, including 25 central incisors, 56 lateral incisors, and 17 canines. Another inclusion criterion was patients having adjacent or contralateral teeth that are intact and restoration free as control. The Hospital Ethics Committee approved the protocol and consent was obtained from every patient after a full explanation of the experiment. The ISSCs were divided into 5 groups according to the abutment and coping material combinations: zz for zirconia abutment with zirconia coping, tz for titanium abutment with zirconia coping, gz for gold/gold hue abutment with zirconia coping, tm for titanium abutment with metal coping, and gm for gold/gold hue abutment with metal coping (Table 1).

### 2.3. Spectrophotometric Assessment of Teeth

The sterilizable adapter of the spectrophotometer's intraoral camera was positioned perpendicularly on the alveolar process over the target ISSCs/teeth. Once the crowns were centered orthoradially in the measuring square depicted on the LED screen, spectrophotometric data were recorded three times consecutively. The device also had a build-in aiming routine that enabled reproducible positioning perpendicular to the facial teeth surface to ensure equal measurement conditions for all teeth evaluated. Prior to every measuring cycle, the spectrophotometer should be calibrated using a white and green ceramic tile supplied by the manufacturer.

### 2.4. Color and Translucency Assessment of Implant Crowns and Teeth

**Figure 1:**
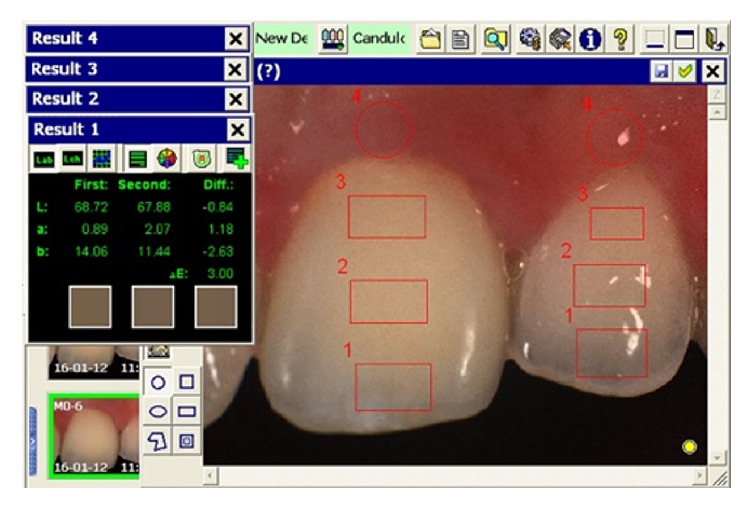
Numbers 1, 2, and 3 represent the crown/tooth areas and number 4 shows the peri-implant mucosa/gingival areas. These areas were used for measurement of color differences Δ*E*_*i*_, Δ*E*_*b*_, Δ*E*_*c*_, and Δ*E*_*g*_, respectively.

All images were transferred to the computer and the shade determination was executed using unique software from the manufacturer. Spectrophotometric evaluation of the color difference Δ*E* between the implant crown and the adjacent/contralateral tooth was measured in 3 areas [5]: incisal third (Δ*E*_*i*_), 3.0 × 1.5 mm; body third (Δ*E*_*b*_), 3.0 × 1.5 mm; and cervical third (Δ*E*_*c*_), 3.0 × 1.5 mm (Figure 1).

Determination of Δ*E* was based on the following equations [16]: Δ*L*^*∗*^ = *L*^*∗*^_tooth_ − *L*^*∗*^_crown_, Δ*a*^*∗*^ = *a*^*∗*^_tooth_ − *a*^*∗*^_crown_, Δ*b*^*∗*^ = *b*^*∗*^_tooth_ − *b*^*∗*^_crown_, and Δ*E* = {Δ*L*^*∗*^^2^ + Δ*a*^*∗*^^2^ + Δ*b*^*∗*^^2^}^1/2^. *L*^*∗*^ is lightness, *a*^*∗*^ is chrome along the red-green axis, and *b*^*∗*^ is chrome along the yellow-blue axis.

Δ*E*_*i*_, Δ*E*_*b*_, and Δ*E*_*c*_ were pooled together to calculate the total crown color difference between implant crown and adjacent/contralateral tooth (Δ*E*_*t*_) for each pair. A critical threshold of Δ*E* (3.7) for intraoral color distinction by the naked eye was considered [17, 18].

Additionally, the difference between crown/tooth color on white and black background was calculated as the translucency parameter (TP), at incisal, body, and cervical part, respectively, with TP = {(*L*^*∗*^_*B*_ – *L*^*∗*^_*W*_)^2^ + (*a*^*∗*^_*B*_ – *a*^*∗*^_*W*_)^2^ + (*b*^*∗*^_*B*_ – *b*^*∗*^_*W*_)^2^}^1/2^.

### 2.5. Color Assessment of Peri-Implant and Natural Tooth Mucosa

Spectrophotometric measurements were also used to assess the color difference between the peri-implant soft tissue and the gingival margin of the neighboring/contralateral tooth. Two standardized circular measuring areas (1.5 mm marginally) were positioned over the same part of the soft tissue margin at the top of the gingival zenith at both the implant crown and adjacent/contralateral tooth (Figure 1). The computer software of the spectrophotometer calculated the color difference in these areas and the final Δ*E*_*g*_ was the average of the three comparisons for each pair.

### 2.6. Subjective, Clinical Evaluation of Color

The subjective clinical color evaluation was based on Copenhagen Index Score (CIS) introduced by Dueled et al. 2009 [19].

The color match score of crowns of CIS was used: score 1 was excellent color and not easy to distinguish from the natural, neighboring teeth. Score 2 was satisfactory, almost optimal but the reconstruction differed from the natural, neighboring teeth. Score 3 was moderate, suboptimal color, and score 4 was poor color match. According to the clinical guidelines, crowns with score 3 or 4 were below standard for cementing.

In addition, the clinical evaluation of peri-implant mucosa color was based on the mucosal discoloration score of the CIS: score 1 was when no mucosal discoloration was visible. Score 2 was given for light grayish mucosal discoloration, score 3 was given for a distinct grayish mucosal discoloration, and score 4 was used when metal/abutment was visible.

### 2.7. Statistical Analysis

Descriptive and One-Way ANOVA were used to compare the differences among groups. Univariate ANOVA was used to assess the impaction of different abutment and coping materials. Spearman correlation was used to test the relationships. *p* < 0.05 was considered statistically significant. For these statistical analyses, the SPSS 17.0 software was used.

## 3. Results

The descriptive analysis showed the color difference of the incisal part (2.9 ± 0.3) and body part (2.9 ± 0.4) of the crown with the gold abutment and zirconia coping combination (gz) were unperceptive (<3.7), while all the other measurements were beyond the threshold. Irrespective of the material, the crown color difference from the contralateral teeth increased from the incisal part to the cervical part, but the difference among the three parts did not reach a significant level. The average total crown color difference (mean Δ*E*_*t*_, 4.7 ± 0.1) was close to that of the body part (mean Δ*E*_*b*_, 4.7 ± 0.2) (Table 2).

**Figure 2:**
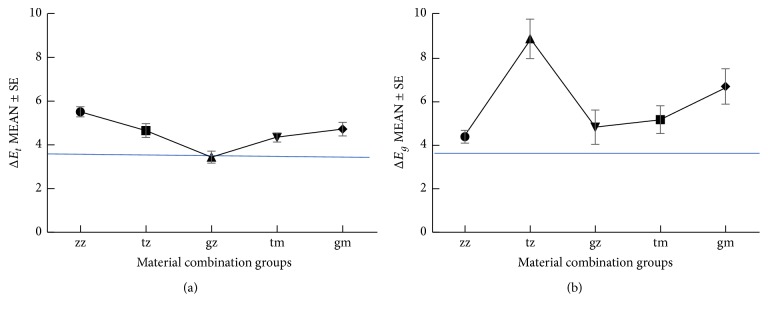
(a) demonstrates the effect of different restorative material combinations on the color of the implant crown. The gz group showed the smallest color change (Δ*E*_*t*_: 3.4 ± 0.3), and the zz group showed the biggest (Δ*E*_*t*_: 5.5 ± 0.2). (b) demonstrates the effect of different restorative material combinations on the color of the peri-implant mucosa. The zz group showed the smallest color change (Δ*E*_*g*_: 4.3 ± 0.2), and the tz group showed the biggest (Δ*E*_*g*_: 8.8 ± 0.9).

The combination of gold abutment with zirconia coping (gz, 3.4 ± 0.3) induced significantly less crown color difference than the all-ceramic group (zz, 5.5 ± 0.2, *p* = 0.001) and the titanium abutment with zirconia coping (tz, 4.6 ± 0.3, *p* = 0.040), as well as gold abutment with metal coping (gm, 4.7 ± 0.3, *p* = 0.022). The all-ceramic implant crowns (zz) demonstrated significantly more color difference than porcelain-fused-to-metal (PFM) restorations (tm + gm, *p* = 0.007). Thus, the combination of gold/gold hue abutment with zirconia crown coping (gz) was tested to have a more acceptable crown color match than the other groups (Figure 2(a)).

As for the peri-implant soft tissue/gingiva, all the color differences measured were beyond the threshold (>3.7), in which the all-ceramic (zz, 4.3 ± 0.2) combination was the smallest, and the titanium abutment with zirconia coping (tz, 8.8 ± 0.9) was the biggest (Table 2). Both the abutment material (*p* = 0.001) and the abutment-coping interaction (*p* = 0.001) could affect the color appearance of the peri-implant mucosa (Table 2). The mucosal discoloration was significantly smaller in all-ceramic (zz) combination compared with titanium abutment-zirconia crown coping (tz), while no significant difference was found between the zz and gz groups. In addition, significant difference was found between the all-ceramic group (zz) and the PFM restorations (tm + gm, *p* = 0.019), demonstrating that all-ceramic (zz) restorations were the more acceptable choice for an esthetic peri-implant mucosa (Figure 2(b)).

Table 3 demonstrates the translucency parameter (TP) decreased from the incisal part to the cervical part at both the implant crown and natural teeth. There was no significant difference of TP among material groups at the incisal and the cervical part and the TP of implant crowns were significantly lower than that of natural teeth. As for the body part of the crown, the TP of all-ceramic group was the highest among material groups and the TP of all material groups were significantly lower than that of the natural teeth.

**Figure 3:**
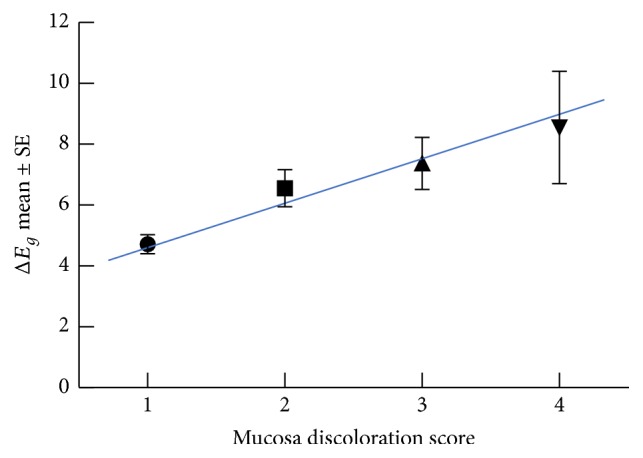
The Spearman correlation between Δ*E*_*g*_ and the mucosal discoloration score, with *r*_*s*_ = .379 and *p* = 0.001.

Regarding subjective clinical evaluation of crown color match score and gingival discoloration score, there was no significant difference between all-ceramic restorations and PFM restorations.  Figure 3 demonstrates a significant correlation between the spectrophotometric gingival color difference (Δ*E*_*g*_) and the mucosal discoloration score, with *r*_*s*_ = 0.379 and *p* = 0.001. However, no significant correlation was found between the spectrophotometric crown color difference (Δ*E*_*t*_) and the crown color match score.

Table 4 demonstrated a descriptive analysis of the shade parameters for natural teeth. These results demonstrated a characteristic increase of *L*, *a*, and *b* from the incisal part to the cervical part of the central and lateral incisors. It could also be noticed that canines compared to central incisors had lower *L* and higher *a* and *b* values.

## 4. Discussion

The present study showed the ideal material combination for implant crown color match was gold/gold hue abutment with zirconia crown coping, while the all-ceramic combination demonstrated a reversely big spectrophotometric color difference.

In our study, the zirconia coping material was tested to have no improvement on crown color match spectrophotometrically. It can be explained by the fact that the Y-TZP zirconia coping was semitranslucent, and the increased thicknesses of zirconium oxide to intensify the strength would compromise the total esthetic result. Vichi [8] reported that the white color of the zirconia and its low translucency still limit the possibility of imitating the natural appearance of a tooth and requires layering with feldspathic ceramic. Additionally, compared with zirconium abutment that gives a white color, the high-gold alloy abutment was reported to have a brighter and yellowish color shift in restoration [20, 21], which may develop a more similar color to natural teeth dentin. These gave the explanation to why the all-ceramic restoration was not ideal as expected as for the aesthetic appearance of the restoration in our study.

Besides lightness, hue, and chrome, the optical characteristics of natural teeth include varying degrees of translucency and opacity, as well as opalescence, iridescence, and fluorescence, of which the translucency was considered important to restoration. It could be reflected by transmittance (T) directly or contrast ratio (CR) and translucency parameter (TP) indirectly. Soler et al. [22] reported that the TP at incisal part of the natural upper central incisors was 4.8–6.5 by Spectroshade; our study gave the similar value. Hasegawa et al. [23] demonstrated the value of TP was highest at the incisal site and decreased in the direction of the root. Our study came to the same conclusion. Another study [24] compared the TP at incisal, body, and cervical parts of three post-core materials with the same zirconia crown demonstrating that the body part of the all-ceramic crown showed a better translucency than the other materials. And our study drew the same conclusion. All-ceramic combination is better than other material combinations, but still not as matching to natural teeth as expected. That might be because the Y-TZP was initially considered as an opaque material and limited translucency was a major drawback of Y-TZP restorations [25].

Our study also demonstrated that the all-ceramic combination induced the smallest spectrophotometric mucosal discoloration and the titanium abutment with zirconium coping combination induced the biggest discoloration. In a recently published study by Bressan et al. [14], the sequence of tz > gz > zz regarding the gingival color discoloration was reported, which is in accordance with the results of our study. In addition, all-ceramic restorations revealed a significantly better color match to the unrestored neighboring teeth than PFM restorations on titanium or gold abutments in Jung et al.'s research [15], as it was proved in the present study.

There was significant correlation between the mucosal discoloration score and the spectrophotometric color difference between the peri-implant mucosa and the natural gingiva, which demonstrated the validation of the subjective index score of mucosal discoloration score. However, no significant correlation was found between the crown color match score and the total spectrophotometric color difference in our study. Some investigators found a significant correlation between instrumental measurements and human color perception [26, 27], while others reported no significant agreement [28, 29]. The reason may be that other factors such as implant crown volume, outline, translucency, and characterization also affect people's clinical perception during color evaluation [30]. On the other hand, in those studies, the implant crown color was assessed as a whole crown, but in our study, the crown was divided into three parts for color evaluation to adopt the three-part color determination method in clinic. Therefore, the spectrophotometric measurements were not in full accordance with the whole crown color match score. Another possible reason could be that most of Δ*E*_*t*_ values, compared to the Δ*E*_*g*_ values, were much closer to the threshold of 3.7, which made it even more difficult for naked eye to distinguish.

Several studies have been conducted to assure the correct using of the spectrophotometer used in the present study [3, 6, 15]. It was proved that this device can eliminate the subjective variations of shade selection by taking two-dimensional image capture of the target tooth. However, the influence of random and systematic errors could not be neglected [31, 32]. Therefore, it was better to compare the target implant crown with the neighboring teeth on the same picture rather than comparing with the contralateral teeth in another picture. This would avoid the deviation between different picturing time and environment, even though the present device has a built-in antifog and positioning program [33]. Additionally, it was reported by Karamouzos et al. [34] that the teeth position (posterior teeth, mandibular incisors) and the mesial and distal areas of natural teeth would affect the long-term repeatability and reproducibility of spectrophotometric measurements, and it was advisable to take particular precautions during the measuring process on the curved surfaces of posterior teeth as well as the labial surfaces of lower anterior teeth. Chu et al. [5] pointed out that both instrumental and visual color matching methods should be used, as they complement each other and can lead towards predictable aesthetic outcome.

This study was a clinical retrospective study. It did not take the veneering ceramic material into consideration, although the outer porcelain was considered more translucent and allowed the zirconia core material color to show [35]. Another limitation was it did not take the coping thickness and veneering staining into account for crown color, neither did the mucosa thickness for gingival color evaluation. Thus, further studies are needed.

## 5. Conclusion

Within the limitation of study, a gold/gold hue abutment with zirconia crown coping was the best choice for an esthetic crown and the all-ceramic combination was the best for peri-implant gingival.

## Figures and Tables

**Table 1:** Materials of abutments and crown copings.

Abutment	
z	Zirconia
t	Titanium
g	Gold alloy: cast-to abutment (Astra Tech): Au 60%, Pt 19%, Pd 20%, Ir 1%, or gold-hue titanium
Veneering	IPS Empress 2: apatite glass-ceramic (Ivoclar Vivadent)
	IPS d.SIGN: fluorapatite leucite glass-ceramic (Ivoclar Vivadent, Schaan, Liechtenstein)
Crown coping	
z	Procera Zirconia: yttria-stabilized tetragonal zirconia polycrystal, Y-TZP (Nobel Biocare™, Gothenburg, Sweden)
m	ORION WX: gold alloy, AU 52.0%, Pd 38.0%, In 8.2%, Ga 1.6%, and Ag and Re < 1% (Elephant Dental BV, Hoorn, Netherlands)

**Table 2:** Color difference (Δ*E*) of the implant crown and peri-implant mucosa compared to neighboring tooth and gingiva (*M* ± SE and *p* values) based on different restorative material combinations.

	Δ*E*_*i*_	Δ*E*_*b*_	Δ*E*_*c*_	Δ*E*_*t*_	Δ*E*_*g*_
Group					
zz (*n* = 40)	4.8 ± 0.3^a^	5.8 ± 0.5^ab^	5.8 ± 0.3^a^	5.5 ± 0.2^ab^	4.3 ± 0.2^ab^
tz (*n* = 10)	4.3 ± 0.3	3.8 ± 0.3^a^	5.8 ± 0.7	4.6 ± 0.3^c^	8.8 ± 0.9^acd^
gz (*n* = 12)	2.9 ± 0.3^ab^	2.9 ± 0.4^b^	4.1 ± 0.4	3.4 ± 0.3^acd^	4.8 ± 0.7^c^
tm (*n* = 21)	4.1 ± 0.3	4.5 ± 0.5	4.3 ± 0.2^a^	4.3 ± 0.2^b^	5.1 ± 0.6^d^
gm (*n* = 15)	5.2 ± 0.4^b^	4.2 ± 0.4	4.6 ± 0.7	4.7 ± 0.3^d^	6.7 ± 0.8^b^
Mean (*n* = 98)	4.4 ± 0.1	4.7 ± 0.2	5.1 ± 0.2	4.7 ± 0.1	5.4 ± 0.2
Material					
Abut.	0.024^*∗*^	0.001^*∗*^	0.228	0.001^*∗*^	0.001^*∗*^
Coping	0.020^*∗*^	0.124	0.382	0.144	0.186
Abut. & coping	0.009^*∗*^	0.645	0.116	0.017^*∗*^	0.001^*∗*^

Same superscript letters indicate significantly different mean values within each column; ^*∗*^*p* < 0.05.

**Table 3:** Translucency measurement (TP) at 3 parts of natural teeth and implant crowns based on material combinations (*M* ± SE).

	TP_*i*_	TP_*b*_	TP_*c*_
Group			
Teeth	7.86 ± 0.3	1.20 ± 0.2	1.01 ± 0.2
zz (*n* = 40)	5.22 ± 0.2	0.81 ± 0.4	0.37 ± 0.3
tz (*n* = 10)	5.09 ± 0.3	0.54 ± 0.2	0.34 ± 0.2
gz (*n* = 12)	5.67 ± 0.3	0.59 ± 0.3	0.41 ± 0.4
tm (*n* = 21)	4.93 ± 0.3	0.47 ± 0.2	0.29 ± 0.2
gm (*n* = 15)	5.02 ± 0.2	0.60 ± 0.3	0.20 ± 0.2

**Table 4:** The color parameters of contralateral/neighboring natural teeth (M ± SE).

Part/position	Central incisor (44)	Lateral incisor (17)	Canine (37)
Incisal			
*L*	66.7 + 0.7	67.6 + 0.5	64.1 + 0.9
*a*	2.0 ± 0.2	2.5 + 0.2	3.1 ± 0.2
*b*	14.0 ± 0.5	14.3 ± 0.6	16.2 ± 0.2
Body			
*L*	73.7 ± 0.6	73.0 ± 0.6	70.9 ± 0.8
*a*	2.7 ± 0.2	3.9 + 0.2	4.3 ± 0.1
*b*	18.0 ± 0.4	20.2 ± 0.6	22.8 ± 0.3
Cervical			
*L*	71.4 ± 0.6	71.1 ± 0.5	69.9 ± 0.5
*a*	6.2 ± 0.2	7.6 ± 0.2	7.2 ± 0.4
*b*	19.3 ± 0.3	20.9 ± 0.8	22.1 ± 0.5
Gingiva			
*L*	51.1 ± 0.6	51.9 ± 0.6	51.2 ± 0.7
*a*	25.1 ± 0.6	26.4 ± 0.9	26.8 ± 0.8
*b*	17.5 ± 0.4	17.2 ± 0.3	20.3 ± 0.6

## References

[1] Belser U. C., Schmid B., Higginbottom F. (2004). Outcome analysis of implant restorations located in the anterior maxilla: a review of the recent literature. *The International journal of oral & maxillofacial implants*.

[2] Fürhauser R., Florescu D., Benesch T., Haas R., Mailath G., Watzek G. (2005). Evaluation of soft tissue around single-tooth implant crowns: the pink esthetic score. *Clinical Oral Implants Research*.

[3] Park S. E., Da Silva J. D., Weber H.-P., Ishikawa-Nagai S. (2007). Optical phenomenon of peri-implant soft tissue. Part I. Spectrophotometric assessment of natural tooth gingiva and peri-implant mucosa. *Clinical Oral Implants Research*.

[4] Vermylen K., Collaert B., Lindén U., Björn A.-L., De Bruyn H. (2003). Patient satisfaction and quality of single-tooth restorations: A 7-year follow-up pilot study in private dental practices. *Clinical Oral Implants Research*.

[5] Chu S. J., Trushkowsky R. D., Paravina R. D. (2010). Dental color matching instruments and systems. Review of clinical and research aspects. *Journal of Dentistry*.

[6] Jung R. E., Sailer I., Hammerle C. H. (2007). In vitro color changes of soft tissues caused by restorative materials. *The International journal of periodontics & restorative dentistry*.

[7] Phillips K., Kois J. C. (1998). Aesthetic peri-implant site development. *The restorative connection J. Dental clinics of North America*.

[8] Vichi A., Louca C., Corciolani G., Ferrari M. (2011). Color related to ceramic and zirconia restorations: a review. *Dental Materials*.

[9] Rompen E., Raepsaet N., Domken O., Touati B., Van Dooren E. (2007). Soft tissue stability at the facial aspect of gingivally converging abutments in the esthetic zone: A pilot clinical study. *Journal of Prosthetic Dentistry*.

[10] Heffernan M. J., Aquilino S. A., Diaz-Arnold A. M. (2002). Relative translucency of six all-ceramic systems. Part II: core and veneer materials. *The Journal of prosthetic dentistry*.

[11] Heydecke G., Schnitzer S., Turp J. C. (2005). The color of human gingiva and mucosa: visual measurement and description of distribution. *J. Clinical oral investigations*.

[12] Carossa S., Lombardo S., Pera P. (2001). Influence of posts and cores on light transmission through different all-ceramic crowns: spectrophotometric and clinical evaluation. *The International journal of prosthodontics*.

[13] Takeda T., Ishigami K., Shimada A. (1996). A study of discoloration of the gingiva by artificial crowns. *The International journal of prosthodontics*.

[14] Bressan E., Paniz G., Lops D., Corazza B., Romeo E., Favero G. (2011). Influence of abutment material on the gingival color of implant-supported all-ceramic restorations: A prospective multicenter study. *Clinical Oral Implants Research*.

[15] Jung R. E., Holderegger C., Sailer I. (2008). The effect of all-ceramic and porcelain-fused-to-metal restorations on marginal peri-implant soft tissue color: a randomized controlled clinical trial. *The International journal of periodontics & restorative dentistry*.

[16] Paul S., Peter A., Pietrobon N., Hämmerle C. H. F. (2002). Visual and spectrophotometric shade analysis of human teeth. *Journal of Dental Research*.

[17] Berns R. (2000). *Principle of Color Technology*.

[18] Munsell A. H. (1923). *A Color Notation*.

[19] Dueled E., Gotfredsen K., Damsgaard M. T., Hede B. (2009). Professional and patient-based evaluation of oral rehabilitation in patients with tooth agenesis. *Clinical Oral Implants Research*.

[20] Seghi R. R., Johnston W. M., O'Brien W. J. (1986). Spectrophotometric analysis of color differences between porcelain systems. *The Journal of Prosthetic Dentistry*.

[21] Crispin B. J., Okamoto S. K., Globe H. (1991). Effect of porcelain crown substructures on visually perceivable Value. *The Journal of Prosthetic Dentistry*.

[22] Soler E., Duran-Sindreu F., Basilio J., Roig M., Ardu S., Mayoral J. R. (2017). In vivo and in vitro spectrophotometric evaluation of upper central incisors before and after extraction. *Clinical Oral Investigations*.

[23] Hasegawa A., Ikeda I., Kawaguchi S. (2000). Color and translucency of in vivo natural central incisors. *Journal of Prosthetic Dentistry*.

[24] dongfang Wq L., Ying Y. (2011). Influence of different posts and cores on the translucency and color of all-ceramic crown. *J Pract Stomatol*.

[25] Kim M.-J., Ahn J.-S., Kim J.-H., Kim H.-Y., Kim W.-C. (2013). Effects of the sintering conditions of dental zirconia ceramics on the grain size and translucency. *The Journal of Advanced Prosthodontics*.

[26] van der Burgt T. P., ten Bosch J. J., Borsboom P. C. F., Kortsmit W. J. P. M. (1990). A comparison of new and conventional methods for quantification of tooth color. *The Journal of Prosthetic Dentistry*.

[27] Bolt R. A., Ten Bosch J. J., Coops J. C. (1994). Influence of window size in small-window colour measurement, particularly of teeth. *Physics in Medicine and Biology*.

[28] Okubo S. R., Kanawati A., Richards M. W., Childress S. (1998). Evaluation of visual and instrument shade matching. *The Journal of Prosthetic Dentistry*.

[29] Wang X., Ge J., Fay R. M. (2005). Comparison of the color of ceramics as measured by different spectrophotometers and colorimeters. *The International journal of prosthodontics*.

[30] Gallucci G. O., Grütter L., Nedir R., Bischof M., Belser U. C. (2011). Esthetic outcomes with porcelain-fused-to-ceramic and all-ceramic single-implant crowns: A randomized clinical trial. *Clinical Oral Implants Research*.

[31] Hugo B., Witzel T., Klaiber B. (2005). Comparison of in vivo visual and computer-aided tooth shade determination. *Clinical Oral Investigations*.

[32] Douglas R. D. (1997). Precision of in vivo colorimetric assessments of teeth. *Journal of Prosthetic Dentistry*.

[33] Chu S. J. (2003). Use of a reflectance spectrophotometer in evaluating shade change resulting from tooth-whitening products. *Journal of Esthetic and Restorative Dentistry*.

[34] Karamouzos A., Papadopoulos M. A., Kolokithas G., Athanasiou A. E. (2007). Precision of in vivo spectrophotometric colour evaluation of natural teeth. *Journal of Oral Rehabilitation*.

[35] Al-Amleh B., Lyons K., Swain M. (2010). Clinical trials in zirconia: a systematic review. *Journal of Oral Rehabilitation*.

